# Association of Influenza Vaccination With Cardiovascular Risk: A Meta-analysis

**DOI:** 10.1001/jamanetworkopen.2022.8873

**Published:** 2022-04-29

**Authors:** Bahar Behrouzi, Deepak L. Bhatt, Christopher P. Cannon, Orly Vardeny, Douglas S. Lee, Scott D. Solomon, Jacob A. Udell

**Affiliations:** 1 Institute of Health Policy, Management, and Evaluation and Temerty Faculty of Medicine, University of Toronto, Toronto, Canada; 2 ICES, Toronto, Canada; 3 Cardiovascular Division, Department of Medicine, Women’s College Hospital, Toronto, Canada; 4 Cardiovascular Division, Brigham and Women’s Hospital, Harvard Medical School, Boston, Massachusetts; 5 Department of Medicine, University of Minnesota, Minneapolis VA Health Care System, Minneapolis, Minnesota; 6 Peter Munk Cardiac Centre, University Health Network, Toronto, Canada

## Abstract

**Importance:**

Influenza infection is associated with increased cardiovascular hospitalization and mortality. Our prior systematic review and meta-analysis hypothesized that influenza vaccination was associated with a lower risk of cardiovascular events.

**Objective:**

To evaluate, via an updated meta-analysis, if seasonal influenza vaccination is associated with a lower risk of fatal and nonfatal cardiovascular events and assess whether the newest cardiovascular outcome trial results are consistent with prior findings.

**Data Sources:**

A previously published meta-analysis of randomized controlled trials (RCTs) and a large 2021 cardiovascular outcome trial.

**Study Selection:**

Studies with RCTs published between 2000 and 2021 that randomized participants to either influenza vaccine or placebo/control. Eligible participants were inpatients and outpatients recruited for international multicenter RCTs and randomized to receive either influenza vaccine or placebo/control.

**Data Extraction and Synthesis:**

PRISMA guidelines were followed in the extraction of study details, and risk of bias was assessed using the Cochrane Collaboration tool. Trial quality was evaluated using Cochrane criteria. Data were analyzed January 2020 and December 2021.

**Main Outcomes and Measures:**

Random-effects Mantel-Haenszel risk ratios (RRs) and 95% CIs were derived for a composite of major adverse cardiovascular events and cardiovascular mortality within 12 months of follow-up. Where available, analyses were stratified by patients with and without recent acute coronary syndrome (ACS) within 1 year of randomization.

**Results:**

Six published RCTs comprising a total of 9001 patients were included (mean age, 65.5 years; 42.5% women; 52.3% with a cardiac history). Overall, influenza vaccine was associated with a lower risk of composite cardiovascular events (3.6% vs 5.4%; RR, 0.66; 95% CI, 0.53-0.83; *P* < .001). A treatment interaction was detected between patients with recent ACS (RR, 0.55; 95% CI, 0.41-0.75) and without recent ACS (RR, 1.00; 95% CI, 0.68-1.47) (*P* for interaction = .02). For cardiovascular mortality, a treatment interaction was also detected between patients with recent ACS (RR, 0.44; 95% CI, 0.23-0.85) and without recent ACS (RR, 1.45; 95% CI, 0.84-2.50) (*P* for interaction = .006), while 1.7% of vaccine recipients died of cardiovascular causes compared with 2.5% of placebo or control recipients (RR, 0.74; 95% CI, 0.42-1.30; *P* = .29).

**Conclusions and Relevance:**

In this study, receipt of influenza vaccination was associated with a 34% lower risk of major adverse cardiovascular events, and individuals with recent ACS had a 45% lower risk. Given influenza poses a threat to population health during the COVID-19 pandemic, it is integral to counsel high-risk patients on the cardiovascular benefits of influenza vaccination.

## Key Points

**Question:**

Is seasonal influenza vaccination associated with lower rates of adverse cardiovascular events?

**Findings:**

In this meta-analysis of 6 randomized clinical trials including 9001 adults who were randomized to influenza vaccination vs matching placebo or standard care, 3.6% of vaccinated patients developed a major adverse cardiovascular event within 12 months compared with 5.4% of those who received placebo or control, a 1.8% significant difference translating into a number needed to vaccinate of 56 patients to prevent 1 event. Higher-risk patients with recent acute coronary syndrome had 45% reduced risk.

**Meaning:**

These results suggest that clinicians and policy makers should continue to counsel high-risk patients on the cardiovascular benefits of seasonal influenza vaccination.

## Teaser

This meta-analysis updates a previous meta-analysis examining the association of seasonal influenza vaccination with prevention of adverse cardiovascular events for a new large clinical trial.

## Introduction

Viral respiratory infections, including those due to the influenza virus, increase the risk for pneumonia and systemic illness that can precipitate fatal and nonfatal cardiovascular events.^1,2^ Underlying cardiovascular disease is also a risk factor for influenza infection, downstream cardiopulmonary complications, and mortality from respiratory infections.^3^ In a prior systematic review and meta-analysis, we found that influenza vaccination was associated with a lower risk of fatal and nonfatal cardiovascular events within a year. A larger risk reduction was seen in patients with recent acute coronary syndrome (ACS).^4^ In this study, we assessed whether new randomized trial data of influenza vaccination from the Influenza Vaccination After Myocardial Infarction (IAMI) trial^5^ was consistent with the findings of our prior meta-analysis and provided further refinement of the cardiovascular risk reduction associated with influenza vaccination.

## Methods

Our analyses focused on published (between 2000 and 2021) randomized clinical trials (RCTs) comparing influenza vaccination with either placebo or control and collecting cardiovascular-related outcomes as primary and/or secondary (including safety) end points. Trial data were included per the Cochrane Collaboration and the Preferred Reporting Items for Systematic Reviews and Meta-Analyses (PRISMA) reporting guideline.

Levels of influenza activity, estimated according to the Centers for Disease Control and Prevention and World Health Organization reports, were categorized as without activity, sporadic, local, regional, and/or widespread.^6^ Risk of bias for each included trial was evaluated by the method of randomization; allocation concealment; patient, investigator, and outcome assessor masking; outcome reporting and ascertainment; and other potential sources of bias as recommended by the Cochrane Collaboration.^7^ Trial quality was determined as high quality by the Cochrane criteria if at least the first 3 criteria were accounted for, low quality if any aspect of the first 3 criteria was unaccounted for, or of uncertain risk of material bias.

### Statistical Analysis

A random-effects Mantel-Haenszel model was used to calculate summary risk ratios (RRs), absolute risk reduction (ARR), and 95% CIs, which used a weighting scheme that depends on the effect measure being used. Our primary outcome was a composite of major adverse cardiovascular events (ie, cardiovascular death or hospitalization for myocardial infarction, unstable angina, stroke, heart failure, or urgent coronary revascularization) within 12 months of follow-up. If unavailable, nonfatal and fatal myocardial infarction and stroke events were used. Our secondary outcome was cardiovascular mortality within 12 months of follow-up. The threshold for significance was *P* < .05 in 2-sided tests. If an outcome achieved statistical significance, the number needed to treat (NNT) to avoid 1 event were derived from the inverse of the pooled estimated ARR. Where available, analyses were stratified by patients with and without recent ACS within 1 year of randomization. Statistical analyses were performed with RevMan version 5.4.1 (Cochrane Training).

## Results

**Figure 1.**
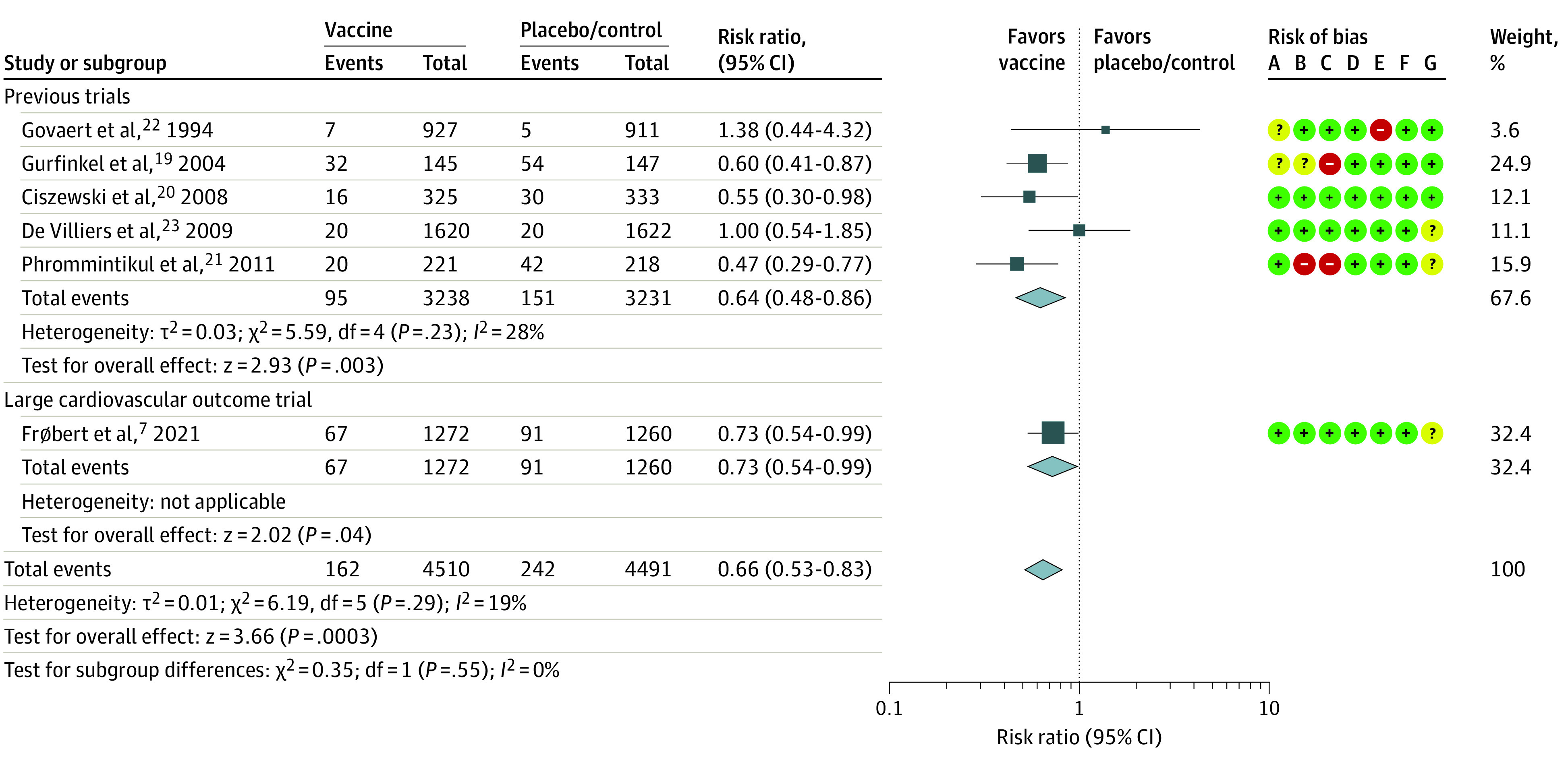
Major Adverse Cardiovascular Events for Influenza Vaccine vs Control When Comparing 2021 Large Cardiovascular Outcome Trial With Previous Meta-analysis Square data markers represent risk ratios; horizontal lines, 95% CIs, with marker size reflecting the statistical weight of the study using random-effects meta-analysis. Diamond data markers represent each subgroup and overall risk ratio with 95% CIs for the outcome of interest. Evaluated using the random-effects Mantel-Haenszel test. Heterogeneity variance τ^2^ calculated using the DerSimonian-Laird estimator. Risk of bias evaluated using standard Cochrane criteria: A, random sequence generation (selection bias); B, allocation concealment (selection bias); C, masking of participants and personnel (performance bias); D, masking of outcome assessment (detection bias); E, incomplete outcome data (attrition bias); F, selective reporting (reporting bias); G, other bias. Red indicates high risk of bias, yellow indicates unclear risk of bias, and green indicates low risk of bias.

In a total of 6 published RCTs, 2890 patients were randomly assigned to receive an intramuscular injection of standard influenza vaccination, 1620 to receive an intranasal live attenuated vaccine, 2504 to receive intramuscular placebo, 1622 to receive intranasal placebo, and 365 to receive no treatment (Table). A total of 9001 participants (mean age, 65.5 years; 3828 women [42.5%]; 4704 participants [52.3%] with a cardiac history) were followed up for a mean duration of 9 months (range, 0.1-12.2 months). Half of the trials were conducted with rigorous randomization, allocation concealment, and masking that met the Cochrane criteria for high quality (ie, low risk of bias) (Figure 1). The remaining studies were considered of uncertain or low quality.

**Table.** Characteristics of Studies Included in the Meta-analysis

Source	Patient cohort	Age, mean (SD), y^a^	Women, No. (%)	Men, No. (%)	No. with cardiac disease (%)	Follow-up, mean (range), mo	Control therapy	No. in control cohort	Vaccine therapy	No. in intervention cohort	Influenza activity^b^	Trial quality	Region
Efficacy trials (influenza vaccine vs placebo/control)													
Gurfinkel et al,^8^ 2004	Inpatients with ACS or outpatients with stable CAD and planned PCI	65 (NR)	62 (20.6)	239 (79.4)	301 (100)	12 (1.0-12.0)	No treatment	147	IM TIV	145	Sporadic	Low	Argentina
Ciszewski et al,^9^ 2008	Outpatients with recent ACS or stable CAD with planned PCI	60 (10)	181 (27.5)	477 (72.5)	658 (100)	9.8 (0.1-12.2)	IM placebo	333	IM TIV	325	Regional	High	Poland
Phrommintikul et al,^10^ 2011	Inpatients with recent ACS	66 (9)	193 (44)	246 (56)	439 (100)	11.8 (0.1-12.0)	No treatment	218	IM TIV	221	Sporadic and widespread	Low	Thailand
Frøbert et al,^5^ 2021	Inpatients and outpatients with recent ACS, coronary angiography or PCI, or stable CAD (high-risk)	59.9 (11.2)	462 (18.2)	2070 (81.8)	2532 (100)	12 (NR)	IM placebo	1260	IM TIV and IM QIV	1272	Sporadic, local, regional, and widespread	High	Sweden, Denmark, Norway, Latvia, UK, Czechia, Bangladesh, Australia
Safety trials (influenza vaccine vs placebo/control)													
Govaert et al,^11^ 1994	Outpatients	67 (NR)	969 (52.7)	869 (47.3)	249 (13.5)	5.0 (2.5-5.0)	IM placebo	911	IM QIV	927	Regional	Uncertain	The Netherlands
De Villiers et al,^12^ 2009	Outpatients	70 (7)	1961 (60.5)	1281 (39.5)	525 (16.2)	8.0 (0.1-8.0)	INL placebo	1622	INL LAIV	1620	Sporadic	High	South Africa

Abbreviations: ACS, acute coronary syndrome; CAD, coronary artery disease; INL, intranasal; IM, intramuscular; LAIV, live attenuated influenza vaccine; NR, not reported; PCI, percutaneous coronary intervention; QIV, quadrivalent inactivated influenza vaccine; TIV, trivalent inactivated influenza vaccine.

^a^ Some results are without SD due to the mean data derived from distribution of participants within age categories or group means being reported without SD.

^b^ Sporadic describes isolated laboratory-confirmed influenza cases or a laboratory-confirmed outbreak in 1 institution, with no increase in activity. Local describes increased incidence of influenza-like illness (ILI), or less than 1 institutional outbreak of ILI or laboratory-confirmed influenza in 1 region with recent laboratory evidence of influenza in that region; virus activity no greater than sporadic in other regions. Regional describes outbreaks of ILI or laboratory-confirmed influenza in more than 1 region with a combined population of less than 50% of the state's total population. Widespread describes outbreaks of ILI or laboratory-confirmed influenza in more than 50% of the regions in the state.

**Figure 2.**
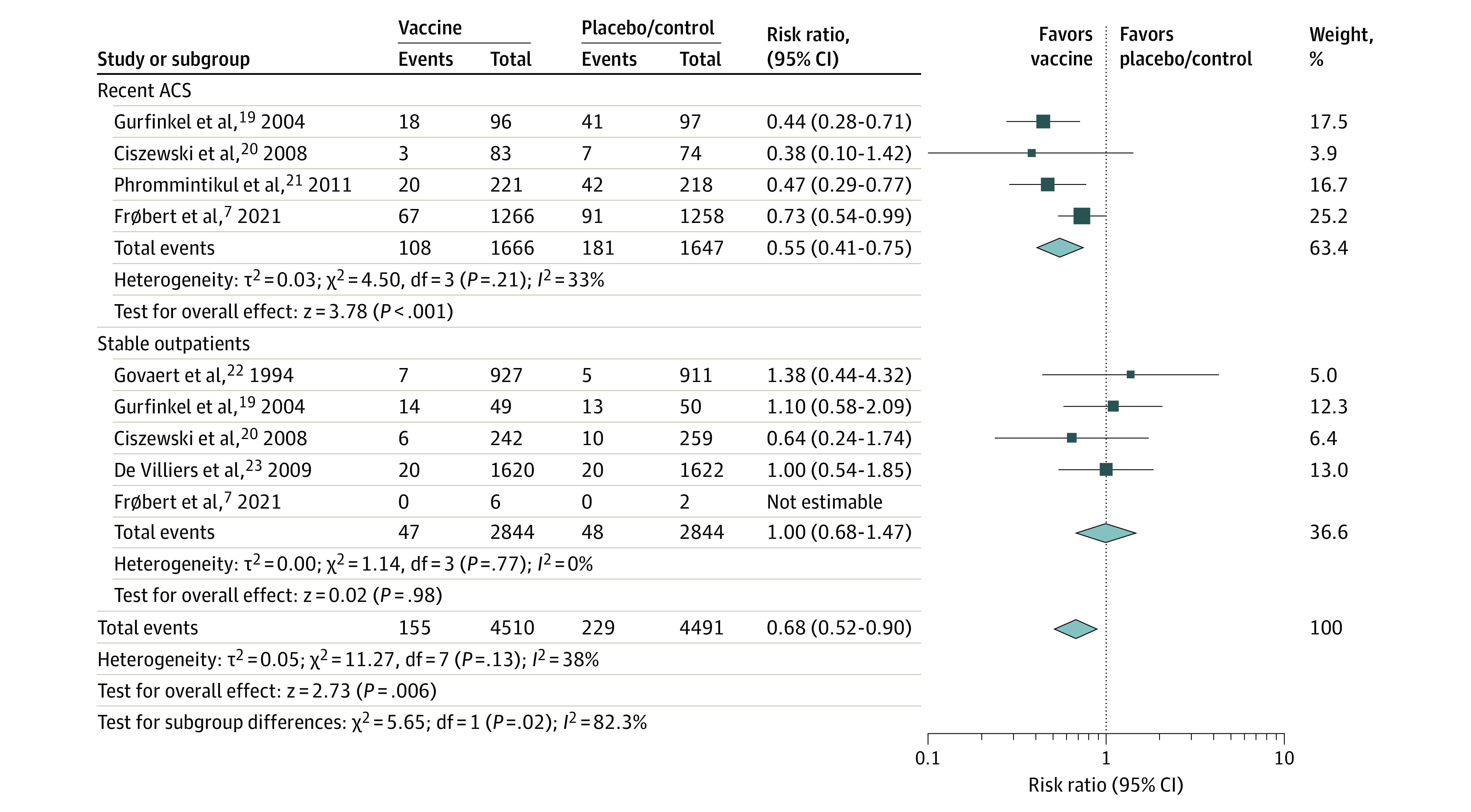
Major Adverse Cardiovascular Events Comparing Influenza Vaccine vs Control Stratified by History of Recent Acute Coronary Syndrome (ACS) Square data markers represent risk ratios; horizontal lines, the 95% CIs with marker size reflecting the statistical weight of the study using random-effects meta-analysis. Diamond data markers represent each subgroup and overall risk ratio and 95% CIs for the outcome of interest. Evaluated using the random-effects Mantel-Haenszel test. Heterogeneity variance τ^2^ calculated using the DerSimonian-Laird estimator.

Among the 4510 patients who received influenza vaccine, 162 patients (3.6%) developed a major adverse cardiovascular event compared with 242 (5.4%) of the 4491 patients who received placebo or control within 1 year of follow-up (RR, 0.66; 95% CI, 0.53-0.83; *I^2^* = 19%; *P* < .001) (Figure 1). This association represented an ARR of 1.8% (95% CI, 0.9%-2.7%; *P* < .001) or an NNT of 56 patients (95% CI, 38-107) to prevent 1 cardiovascular event. A significant treatment interaction was detected in a subgroup analysis of patients with recent ACS (3313 patients; 6.5% vaccine vs 11% placebo/control; RR, 0.55; 95% CI, 0.41-0.75; *I^2^* = 33%; *P* < .001) and stable outpatients (5688 patients; 1.7% for both vaccine and placebo/control; RR, 1.00; 95% CI, 0.68-1.47; *I^2^* = 0%; *P* = .98; *P* for interaction = .02) (Figure 2). For patients vaccinated with a recent ACS, the ARR was 4.5% (95% CI, 2.6%-6.4%; *P* < .001) or an NNT of 23 patients (95% CI, 16-39 patients) to prevent 1 cardiovascular event.

**Figure 3.**
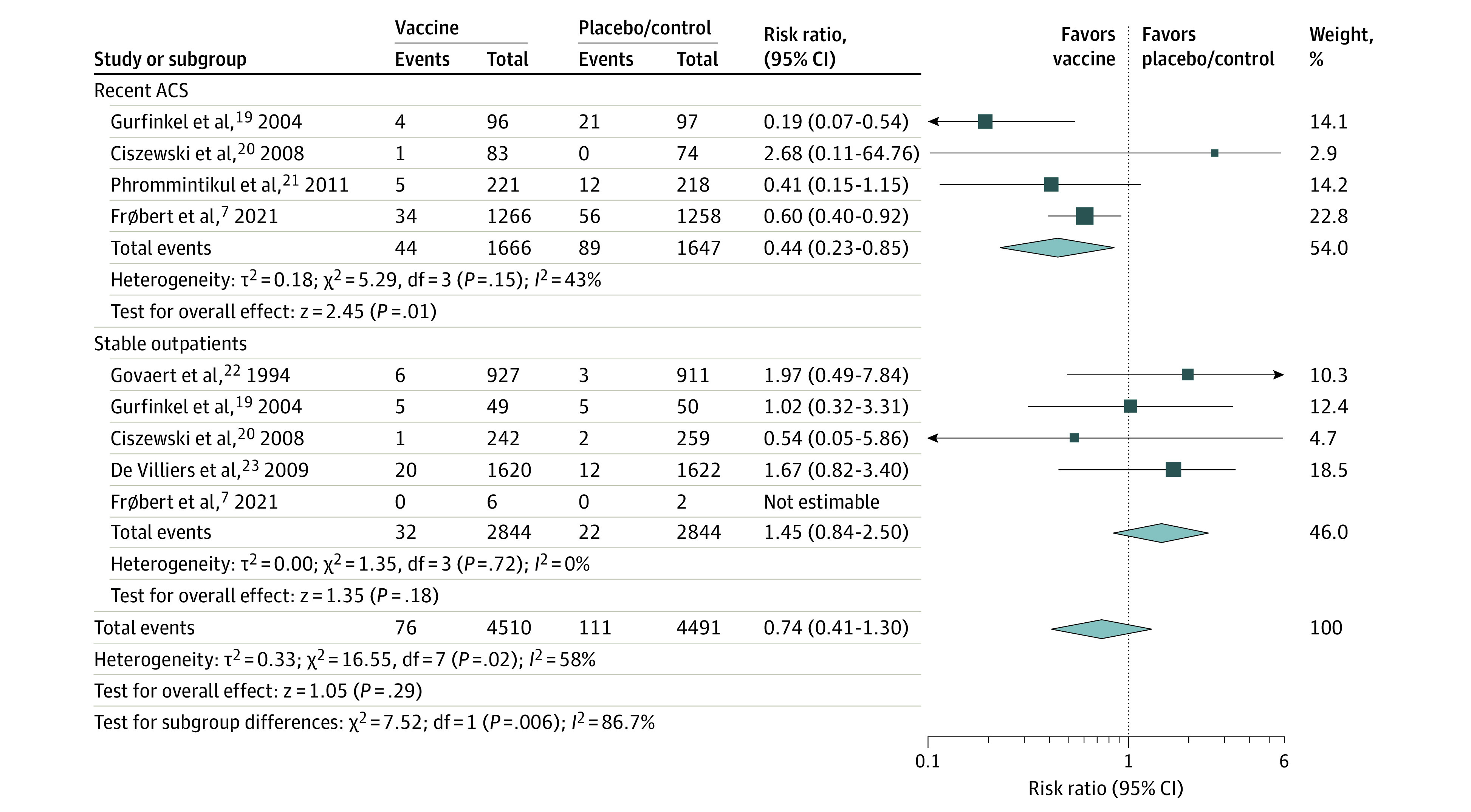
Cardiovascular Mortality Comparing Influenza Vaccine vs Control Stratified by History of Recent Acute Coronary Syndrome (ACS) Square data markers represent risk ratios; horizontal lines, the 95% CIs with marker size reflecting the statistical weight of the study using random-effects meta-analysis. Diamond data markers represent each subgroup and overall risk ratio and 95% CIs for the outcome of interest. Evaluated using the random-effects Mantel-Haenszel test. Heterogeneity variance τ^2^ calculated using the DerSimonian-Laird estimator.

Furthermore, 76 of the 4510 patients who received influenza vaccine (1.7%) died due to cardiovascular causes compared with 111 of the 4491 patients (2.5%) who received placebo or control within 1 year of follow-up, although this result was not significant (RR, 0.74; 95% CI, 0.42-1.30 *I^2^* = 62%; *P* = .29). However, in a subgroup analysis of patients with recent ACS (3313 patients; 2.6% vaccine vs 5.4% placebo/control; RR, 0.44; 95% CI, 0.23-0.85; *I^2^* = 43%; *P* = .01) and stable outpatients (5688 patients; 1.1% vaccine vs 0.8% placebo/control; RR, 1.45; 95% CI, 0.84-2.50; *I^2^* = 0%; *P* = .18), a significant treatment interaction was found (*P* for interaction = .006) (Figure 3). Therefore, for recent ACS, the ARR was 2.8% or an NNT 36 (95% CI, 15-100) patients to prevent 1 cardiovascular death.

## Discussion

Our prior meta-analysis underpinned the need for a large multicenter trial, powered for cardiovascular outcomes, to confirm our findings. Subsequently, the IAMI trial^5^ randomized 2532 patients with recent myocardial infarction to influenza vaccine or placebo and showed a lower risk of composite cardiovascular events. Although the study was terminated early because of the COVID-19 pandemic, with approximately 60% of planned randomization, IAMI (hazard ratio, 0.72) prospectively confirmed our meta-analysis (RR, 0.64) while reducing the percentage of variation across the included studies because of heterogeneity (*I^2^*) to 19%. Another recent outcome trial, Influenza Vaccine to Effectively Stop Cardio Thoracic Events and Decompensated Heart Failure,^13^ demonstrated no difference in efficacy between a high-dose trivalent vs a standard-dose quadrivalent vaccine in patients with recent hospitalization for heart failure or myocardial infarction. However, the lack of a placebo arm limited its incorporation here.

With the addition of the most recent RCT data, we now also observe a significant interaction between the benefits of influenza vaccination for reducing cardiovascular mortality based on underlying cardiovascular risk. Specifically, among patients with a recent ACS, the risk reduction of cardiovascular death is over 50% among those who received seasonal influenza vaccine. The effect sizes reported here for major adverse cardiovascular events and cardiovascular mortality (in patients with and without recent ACS) are comparable with—if not greater than—those seen with guideline-recommended mainstays of cardiovascular therapy, such as aspirin, angiotensin-converting enzyme inhibitors, β-blockers, statins, and dual antiplatelet therapy.^14^

### Limitations

Our study had several limitations. Smaller studies are at risk of selection, performance, or attrition bias, requiring circumspection against overinterpretation. Therefore, it is integral to continue to update future meta-analyses with the results of at least 3 other ongoing large cardiovascular outcome trials (placebo- and active-controlled) that examine various patient populations across the spectrum of cardiovascular disease in other jurisdictions, during contemporary influenza seasons, and using the latest available formulations of seasonal influenza vaccines.^15,16,17^

## Conclusion

Influenza continues to pose a substantial threat to population health during the COVID-19 pandemic, which is why new viral respiratory vaccine research prominently features combination formulations with influenza.^18,19,20^ It is also well established that limitations of the current egg-based mass production systems for seasonal influenza vaccines have curbed the effectiveness of existing vaccines to date.^1^ Alternative vaccine platforms, such as those based in mRNA and other technology, continue to progress toward the end goal of a universal influenza vaccine.^14^ At the same time, patients with cardiovascular disease have also demonstrated an inadequate immune response postvaccination due to processes such as immunosenescence and inflammaging.^1^ Despite potential suboptimal vaccine effectiveness and immune response, the potential risk reduction in major adverse cardiovascular events and cardiovascular mortality with an influenza vaccine is already sizeable. Therefore, it is likely that the forthcoming improved vaccine technologies have the potential to increase this protective benefit.

It is important to evaluate new influenza vaccine platforms for their potential impact on cardiovascular outcomes. Until then, we urge clinicians to continue counselling their high-risk patients on the cardiovascular benefits of seasonal influenza vaccination, especially given the historically low uptake of this low-cost and well-tolerated intervention.^21,22,23^
